# Hearing Assessment after Treatment of Nasopharyngeal Carcinoma with CRT and IMRT Techniques

**DOI:** 10.1155/2015/769806

**Published:** 2015-08-16

**Authors:** Chung-Feng Hwang, Fu-Min Fang, Ming-Ying Zhuo, Chao-Hui Yang, Li-Na Yang, Hui-Shan Hsieh

**Affiliations:** ^1^Department of Otolaryngology, Kaohsiung Chang Gung Memorial Hospital and Chang Gung University College of Medicine, Kaohsiung 83301, Taiwan; ^2^Department of Radiation Oncology, Kaohsiung Chang Gung Memorial Hospital and Chang Gung University College of Medicine, Kaohsiung 83301, Taiwan; ^3^Department of Otolaryngology, Chang Gung Memorial Hospital and Xiamen Medical Center, Fujian 361000, China; ^4^Graduate Institute of Audiology and Speech Therapy, National Kaohsiung Normal University, Kaohsiung 80201, Taiwan

## Abstract

*Objectives*. This study analyzed the long-term hearing loss after treatment of primary nasopharyngeal carcinoma to elucidate its causal factors. *Methods*. Ninety-two nasopharyngeal carcinoma patients were treated with radiotherapy or chemoradiotherapy. Pure tone audiometry was performed before the therapy and annually up to 9 years after completing treatment. The hearing thresholds were corrected for age-related deterioration and compared to the results without adjusting for age. *Results*. The mean air and bone conduction threshold with and without correction for age-related deterioration differed significantly 2–9 years after completing radiotherapy (*p* < 0.05). The audiometry results with age correction showed a flattened configuration compared to the results without age correction. The total radiation dose and radiation modality showed a causal relationship with a greater incidence of hearing loss after therapy (*p* < 0.05). There was more deterioration in the air and bone hearing thresholds with conformal radiotherapy than intensity-modulated radiotherapy (*p* < 0.001). A radiation dose >72 cGy resulted in more severe hearing loss than <72 cGy (*p* < 0.05). *Conclusion*. Hearing loss after completing therapy should be corrected for age-related hearing deterioration to reveal the true extent to which the loss is a therapeutic complication. Both the radiation modality used and the dose were significantly associated with hearing loss.

## 1. Introduction

Nasopharyngeal carcinoma (NPC) is often located in the fossa of Rosenmüller near the opening of the Eustachian tube into the nasopharynx. Of these tumors, 95% are undifferentiated squamous cell carcinoma, which is very sensitive to radiotherapy (RT) [1]. Chemoradiotherapy for locoregionally advanced stages III and IV confers significant improvements in both local control and survival rates [2–4].

While RT or chemoradiotherapy is the standard approach for NPC, the radiation and cisplatin ototoxicity inevitably damage the auditory apparatus [5–8]. This may lead to persistent hearing loss (HL), which affects the patients' quality of life. The literature lacks conclusive information on the incidence and characteristics of HL, as well as the associated risk factors in NPC [9]. Some related factors contribute to HL in postirradiated patients, including radiation dose, radiation technique, adjuvant chemotherapy, age, and middle ear effusion [10–12].

Any study of HL may be erroneous if the influence of patient age on hearing status is not considered because age can affect HL [13]. We reviewed the literature and found no reports that have considered age over the course of NPC. Therefore, this study investigated the level of HL, related factors, and the configuration of the mean audiometric curve, correcting the data according to patient age.

## 2. Materials and Methods

### 2.1. Patients

Ninety-two newly diagnosed cases of NPC were treated in a single medical center between June 1994 and July 2010. Audiograms were obtained before RT and at least 12 months of follow-up after completing RT. We enrolled NPC patients who were older than 18 years and had no significant preexisting HL; that is, the average bone conduction hearing threshold levels (0.5–4 kHz) were below 70 dB before RT. Patients who received additional RT for metastatic or recurrent disease, who were exposed to noise, or who received ototoxic medications other than cisplatin were excluded from the study. This study protocol was approved by the institutional review board of Chang Gung Memorial Hospital.

### 2.2. Chemoradiation Modalities

Patients with stage I/IIa were treated with RT alone, while the locally advanced stage IIb-IV patients were treated with chemoradiation therapy. With updates at our institution, the radiation technique has changed from two-dimensional conformal RT (2D CRT) to three-dimensional conformal RT (3D CRT) to intensity-modulated RT (IMRT). The total radiation dose was 59.4–79.2 Gy with a mean dose of 70.86 Gy. Patients with locally advanced disease were given concurrent intravenous cisplatin chemotherapy at a mean dose of 365.57 (range 120–670) mg/m^2^.

### 2.3. Audiometry and Analysis of Age-Related Deterioration Adjustment of Audiometric Data

Audiometry was performed before and after RT at 1-year intervals for up to 9 years. European Committee for Standardization (CEN) data were used to obtain corrected thresholds by subtracting the loss corresponding to age and sex from the auditory threshold obtained at each frequency [14]. For example, for a 50-year-old male, the threshold was 30 dB HL at 250 Hz, compared to 3 dB HL at 250 Hz in a normal 50-year-old male patient; therefore, the corrected hearing level was 27 dB at 250 Hz after eliminating the effect of age-related degeneration. The hearing thresholds of air conduction at 0.25, 0.5, 1, 2, 4, and 8 kHz and bone conduction at 0.5, 1, 2, and 4 kHz were measured and adjusted. The average threshold levels at 0.5, 1, 2, and 4 kHz were used to indicate hearing ability.

### 2.4. Statistical Analysis

The data were analyzed using SPSS ver. 19.0 (SPSS, Chicago, IL). An independent *t*-test was used to determine the correlation between the possible predisposing factors and average hearing threshold deterioration. Univariate and multivariate analyses were performed with a Cox stepwise logistic regression model to identify independent prognostic indicators. A paired *t*-test was used to assess the serial changes in hearing with or without age adjustment. A *p* value less than 0.05 was considered to be statistically significant. All statistical tests were two-sided.

## 3. Results

This study included 182 ears from 92 patients. The patient characteristics and therapeutic modalities are summarized in Table 1. Patient age ranged from 20 to 76 years and the median age was 50.91 years. According to the 6th AJCC staging system, 10 patients (20 eligible ears) were stage I, 39 patients (77 eligible ears) were stage II, 26 patients (52 eligible ears) were stage III, and 17 patients (33 eligible ears) were stage IV.

### 3.1. Differences in HL Severity without and with Adjustment for Age-Related Deterioration

**Figure 1:**
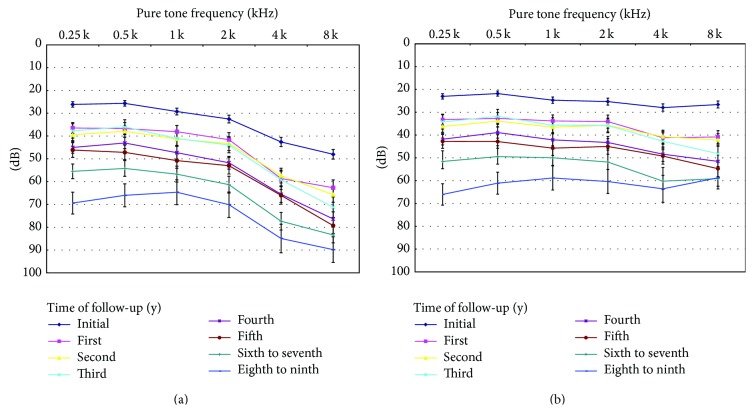
Audiogram of the hearing threshold of all patients before radiotherapy or chemoradiotherapy and after follow-up for 1 to 9 years (◆, ■, ▲, x, *∗*,  ., +, and −, resp.). (a) Without and (b) after adjusting for age-related threshold deterioration (Mean ± SEM).

**Figure 2:**
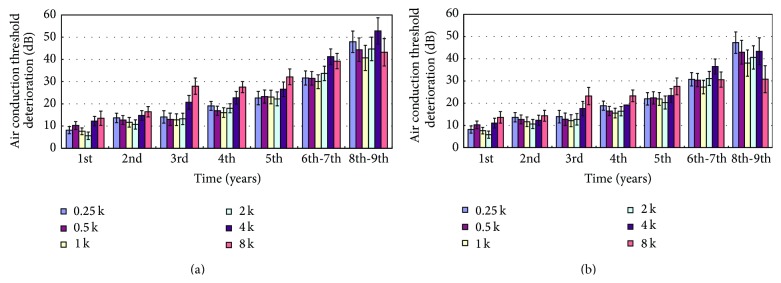
Air conduction threshold deterioration 1 to 9 years after therapy. (a) Without and (b) after adjusting for age-related threshold deterioration (Mean ± SEM).

The average hearing thresholds (not considering age-related HL or presbycusis) are shown in Figure 1(a). The average results, corrected for age-related HL at the time of each audiogram, are shown in Figure 1(b). The hearing thresholds without age correction are approximately equal to hearing thresholds with age correction plus age-related hearing deterioration. The age-related hearing deterioration was especially worse at high frequency. The audiometry results with age-related deterioration correction had a flattened plot compared to results without age correction (Figure 2). The average hearing threshold change at 4 kHz was 42 dB, deteriorating from initial 43 to 85 dB in the 9th year (Figure 1(a)). After the adjustment of age (Figure 2(b)), the average hearing thresholds change at 4 kHz was 36 dB (changed from 28 to 64 dB). Contrastingly, the hearing threshold at 0.5 kHz changed from 26 to 66 dB without adjustment and from 22 to 61 dB with adjustment of age. A better hearing threshold was found after adjustment of age, especially at high frequency.

**Figure 3:**
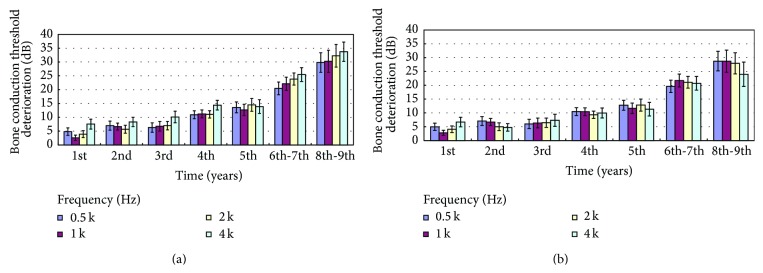
Bone conduction threshold deterioration 1 to 9 years after therapy. (a) Without and (b) after adjusting for age-related threshold deterioration (Mean ± SEM).

In the 9-year follow-up after completing RT, without adjusting for age-related deterioration, the air conduction threshold deteriorated every year (*p* < 0.05), especially at 4–8 kHz (Figure 2(a)). Adjusting for the age-related deterioration in hearing, the air conduction threshold still deteriorated every year at all frequencies (*p* < 0.05). The levels of hearing deterioration were less than those without adjusting (Figure 2(b)). After adjusting for age, the deterioration at high frequencies was lower. Hearing deterioration was progressive and all hearing frequencies appeared to be involved equally. The findings were similar for the bone conduction threshold (Figure 3). The sensorineural hearing threshold deteriorated every year (*p* < 0.05). The deterioration at all frequencies tended to decrease after adjusting for age, especially at 4 kHz (Figure 3(b)). Hearing deterioration without adjustment might be due to both radiation toxicity and aging.

### 3.2. Influence of Adjusting for Age-Related Deterioration on HL

**Figure 4:**
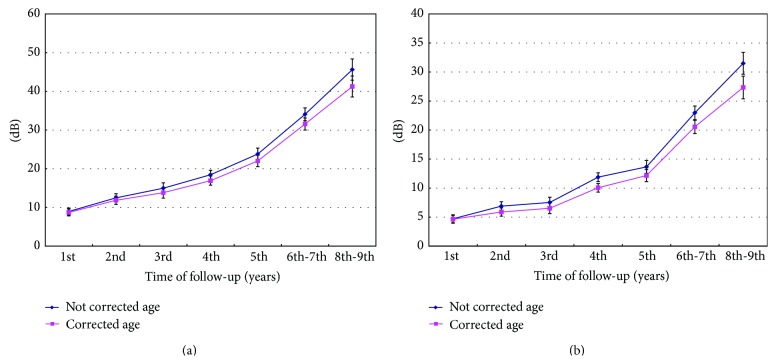
The (a) air and (b) bone conduction hearing deterioration at 0.5–4 kHz with and without age-related correction differed significantly at the 2- to 9-year follow-ups (*p* < 0.05), while the differences were not significant 1 year after completing RT.

The air conduction hearing deterioration at 0.5–4 kHz with and without age-related correction differed significantly at the 2- to 9-year follow-ups (*p* = 0.02 at 2 years; *p* < 0.001 at 3 to 9 years, resp.), while the differences were not significant at 1 year after completing RT (*p* = 0.16) (Figure 4(a)). The patients lost average 46 dB (0.5–4 kHz) without age-related correction 9 years after treatment. The hearing deterioration decreased to 41 dB after age-related correction.  Figure 4(b) also showed significant differences of bone conduction deterioration after age-related correction (*p* = 0.01 at 2 years, *p* = 0.006 at 3 years, and *p* < 0.001 at 4 to 9 years, except *p* = 0.554 at 1 year). We found that adjusting for age decreased the deterioration of HL after completing treatment.

### 3.3. Factors Determining HL after Age-Related Deterioration Adjustment

The mean hearing deterioration after adjusting for age at 0.5–4 kHz in the first 5 years was analyzed in terms of gender, age, radiation modality (2D-3D CRT and IMRT), concurrent chemotherapy, radiation dose (<72 Gy and >72 Gy), and post-RT otitis media with effusion (OME) (Table 2). The radiation modality (24.00 dB versus 12.21 dB *p* < 0.001; 14.01 dB versus 6.85 dB *p* < 0.001), radiation dose (10.24 dB versus 18.69 dB *p* = 0.017; 6.66 dB versus 10.26 dB *p* = 0.026), and OME (15.54 dB versus 19.46 dB *p* = 0.019; 8.09 dB versus 12.50 dB *p* = 0.016) were important determinants of hearing deterioration via both air and bone conduction. Radiation modality (*p* < 0.001) and radiation dose (*p* = 0.015, *p* = 0.004 for both air and bone) remained significant in multivariate analyses. We found that deterioration in both the air and bone conduction thresholds was less in the IMRT group than in the 2D/3D CRT group 5 years after completing RT. The deterioration in hearing threshold was also greater when the total radiation dose to the primary tumor was >72 Gy.

In order to evaluate the effect of cisplatin, we further analyzed the hearing deterioration in both RT (89 ears without cisplatin) and chemoradiotherapy (93 ears with cisplatin) groups. The radiation modality (24.67 dB versus 9.87 dB *p* < 0.001; 16.19 dB versus 5.91 dB *p* < 0.001), radiation dose (6.73 dB versus 15.45 dB *p* = 0.016; 3.60 dB versus 9.81 dB *p* = 0.004), and OME (10.43 dB versus 18.86 dB *p* = 0.027; 5.85 dB versus 12.68 dB *p* = 0.011) were important determinants of hearing deterioration via both air and bone conduction in the chemoradiotherapy group. Radiation modality (*p* < 0.001 for both air and bone) was significant in multivariate analyses. Only radiation dose (12.07 dB versus 22.67 dB *p* = 0.036 for air conduction) remained significant in the RT group.

## 4. Discussion

### 4.1. Hearing Changes after Adjusting for Age-Related HL

The prevalence of presbycusis is 35–50% in those aged 65 years or older; consequently, HL may be excessively attributed to RT in elderly patients [15]. NPC patients, like the general population, undergo the expected age-related hearing threshold shift. In our study, postirradiation audiograms without adjusting for the age-related hearing shift indicated that the hearing deterioration started at high frequencies. The longer the follow-up was, the more extensively the frequencies were affected, including the speech frequencies. Previous studies of HL as an adverse effect of RT have reported a greater incidence of HL at high frequencies compared to speech frequencies [9, 16]. In one study, at least 2 of 10 patients developed HL at speech frequencies and at least 3 of 10 did at frequencies greater than 4 kHz when treated for NPC [6]. Li et al. reported HL in 60% of patients at the speech frequency range and 95% for high-frequency HL [17]. These findings are consistent with our results without adjusting for age.

After adjusting for age, the deterioration at high frequencies was lower in our study. The longer the follow-up was, the less the deterioration at high frequency was found (Figures 2 and 3). There is still secondary deterioration at speech frequencies. The hearing deterioration pattern differed with and without adjusting for age-related deterioration. Without adjusting for the age-related threshold shift, the audiogram plot showed greater deterioration at high frequencies, while the curve was flattened with equal deterioration at all frequencies after making the adjustment (Figure 1).

To elucidate the true HL caused only by therapy or NPC itself, the effects of age-related degeneration should be eliminated by adjusting for age and sex. This led to two important findings. First, RT-induced HL is progressive and all hearing frequencies appear to be damaged equally. Second, high-frequency HL might be due to both radiation toxicity and presbycusis.

Furthermore, the deterioration in air conduction was greater than that of bone conduction because these patients often develop middle ear damage after RT. This finding is consistent with studies that have reported long-term conductive HL caused by Eustachian tube dysfunction and middle ear fibrosis with persistent sensorineural HL, indicating a mixed HL [18–20].

### 4.2. Factors Related to HL

The radiation technique appears to affect the development of HL, although studies are required to verify this hypothesis [9]. We found that deterioration in both the air and bone conduction thresholds was less in the IMRT group than in the 2D/3D CRT group (12.21 dB versus 24.00 dB and 6.85 dB versus 14.01 dB, *p* < 0.001) 5 years after completing RT (Table 2). This is similar to reports that the incidence of HL ranges from 26 to 85% when using a CRT technique [17, 21–23] versus 16% with IMRT [6]. These results give insight into the enhanced normal-tissues-sparing capacity of IMRT. Our study compared CRT and IMRT techniques in a single institute and there was more deterioration in both the air and bone hearing thresholds with CRT than with IMRT.

The deterioration in hearing threshold was greater when the total radiation dose to the primary tumor was >72 Gy (Table 2). These results are consistent with reports that higher radiation doses lead to greater incidence and severity of HL [7, 8, 24]. However, Liberman et al. found no significant difference in the hearing threshold at any frequency when the total mean radiation dose to the primary tumor was 6887 cGy or the mean radiation dose at ear level was 4200.0 cGy, although their study followed 11 patients for 5–10 months [25]. The postirradiation HL usually presents clinically at least 12 months after completing RT [9]. A longer follow-up is needed for an accurate evaluation of HL incidence after RT.

Chemotherapy using cisplatin did not predict HL, in agreement with previous reports (Table 2) [24, 26]. The ototoxicity of cisplatin is dose dependent and the incidence of HL increases with a total dose of 600–1050 mg/m^2^ [27, 28]. In our study, the total dose of cisplatin was less than 600 mg/m^2^, which might explain why the threshold deterioration was not correlated with chemotherapy. In order to evaluate the effect of cisplatin, we further analyzed the hearing deterioration in both RT (89 ears without cisplatin) and chemoradiotherapy (93 ears with cisplatin) groups. The radiation modality (CRT and IMRT), radiation dose, and OME were significant via both air and bone conduction in the chemoradiotherapy group. Radiation modality remained significant in multivariate analyses. Only radiation dose was significant in the RT group. Cisplatin appeared to increase the hearing deterioration in NPC patients using a CRT technique.

Post-RT OME was not an independent predictor of hearing threshold deterioration. The characteristics of post-RT OME differed with the radiation dose and modality. The post-RT OME group treated with IMRT and a radiation dose <72 Gy might have had less severe hearing threshold deterioration. With IMRT, the dose to the Eustachian tube was <52 Gy, reducing the incidence of post-RT OME and severity of air and bone conduction threshold (0.5–4 kHz) deterioration [29, 30]. Young and Hsieh reported that a total radiation dose >70 Gy exacerbated Eustachian tube dysfunction [31].

Some studies have observed a persistent association between the presence of post-RT OME and HL [24, 32]. The presence of middle ear effusion is evidence of Eustachian tube dysfunction as a complication of radiation, with potential inner ear damage [19, 33]. The development of post-RT OME is another manifestation of radiation damage and it indicates individual sensitivity to radiation [24].

We found no significant correlation between age and gender and post-RT HL data adjusted or not for age-related threshold deterioration. The effects of age and gender on the development of RT-induced HL were inconsistent in previous reports [9]. Some authors found that older patients [5, 7, 24] and males [24, 34] were more likely to develop HL after RT. These conclusions may be incorrect, given that the effects of patient age and gender* per se* on hearing status were not considered; again, these factors affect HL.

### 4.3. Limitations

This study was retrospective in nature, and several potential limitations should be mentioned. First, follow-up was performed after various intervals by different physicians, and it is likely that the follow-up protocols differed greatly. For example, the instruments used for measurement will have varied (the time series is very long and audiometers were almost certainly were replaced over time). In addition, the RT techniques will have varied. NPC patients may have died or become lost to follow-up. Second, data incompleteness and differences in the way data were annotated in databases and medical charts render it difficult to define the cause of HL after RT. Moreover, all the evaluation of long-term HL after treatment of primary NPC was performed at a single center. We believe that these factors have affected all cases similarly, merely rendering our estimations imprecise; we do not believe that we have exaggerated or minimized such estimations. Patients with possible confounding factors, such as significant preexisting HL, use of ototoxic medications other than cisplatin, or exposure to noise, were excluded from the study.

There is a concern that the issue of cisplatin, an ototoxic drug, could confound the data collected. Since late stage NPC patients treated with cisplatin were included in this study, it was difficult to separate an aging and radiation in patients with cisplatin versus no cisplatin. Cisplatin interferes with the overall findings and conclusions reached.

## 5. Conclusion

After adjusting for age-related hearing threshold deterioration, HL owing to the treatment of NPC was present clinically at least 12 months after RT and involved all frequencies. Therefore, it is essential to adjust for patient age when determining the true change in hearing following RT or chemoradiotherapy. IMRT and total radiation dose less than 72 Gy tended to result in less deterioration in hearing.

## Supplementary Material

The hearing thresholds of air conduction at 0.25, 0.5, 1, 2, 4, and 8 kHz were measured and adjusted (Supplementary Table 1). The air (0.25–8 kHz) and bone (0.5–4 kHz) conduction hearing deterioration with and without age-related correction at the 1- to 9-year follow-up after RT were listed in Supplementary Table 2 and 3, respectively. The mean air and bone conduction hearing deterioration at 0.5–4 kHz with and without age-related correction were measured (Supplementary Table 4).

## Figures and Tables

**Table 1:** Characteristics of the nasopharyngeal carcinoma patients.

Parameter	Patients (*n* = 92) (%)	Ears (*n* = 182) (%)
Age (y)		
Range	20–76	
Median	50.91	
Sex		
Male	65 (70.75%)	
Female	27 (29.34%)	
Stage		
I	10 (10.86%)	20 (11%)
II	39 (42.39%)	77 (42.31%)
III	26 (28.26%)	52 (28.57%)
IV	17 (18.47%)	33 (18.13%)
Treatment regimen		
RT alone	45 (48.91%)	89 (48.9%)
Chemoradiotherapy	47 (51.08%)	93 (51.1%)
Radiation modalities		
2D CRT	10 (10.86%)	19 (10.44%)
3D CRT	28 (30.43%)	56 (30.77%)
IMRT	54 (58.69%)	107 (58.80%)
Total radiation dose (Gy)		
Range	59.4–79.2	
Mean	70.86	
Cisplatin dose (mg/m^2^)		
Range	120–670	
Mean	363.55	
Postirradiated OME		
Yes		71 (39.00%)
No		111 (61.00%)

^*∗*^OME = otitis media with effusion.

**Table 2:** Factors predicting the average air and bone conduction threshold deterioration at the 1- to 5-year follow-up after RT.

Variables	*N* (= 182)	Air (dB) Mean ± SEM	*p* value	Bone (dB) Mean ± SEM	*p* value
Sex					
Female	53	15.67 ± 3.02	0.556	9.05 ± 12.27	0.591
Male	129	17.64 ± 1.65		10.12 ± 12.05	
Radiation modality					
2D-3D CRT	75	24.00 ± 2.34	0.000	14.04 ± 1.54	0.000
IMRT	107	12.21 ± 1.71		6.85 ± 0.99	
Age (years)					
<50	89	18.73 ± 2.30	0.267	10.55 ± 1.38	0.421
>50	93	15.47 ± 1.81		9.10 ± 1.15	
Treatment regimen					
RT alone	89	19.93 ± 2.23	0.060	10.65 ± 1.23	0.359
Chemoradiotherapy	93	14.32 ± 1.86		9.58 ± 1.30	
Radiation dose					
<72 Gy	35	10.24 ± 3.01	0.017	6.66 ± 1.65	0.026
>72 Gy	147	18.69 ± 1.63		10.26 ± 1.03	
Otitis media effusion					
No	111	15.54 ± 1.81	0.019	8.09 ± 1.00	0.016
Yes	71	19.46 ± 2.43		12.50 ± 1.65	

^*∗*^SEM = standard error of the mean; RT = radiotherapy; 2D-3D CRT = 2-dimensional-3-dimensional conformal radiotherapy; IMRT = intensity-modulated radiotherapy.
