# Supplementary material for: Hearing Assessment after Treatment of Nasopharyngeal Carcinoma with CRT and IMRT Techniques
Source: Biomed Res Int. 2015 Aug 16;2015:769806. doi: 10.1155/2015/769806 (PMC4553178; doi:10.1155/2015/769806)
Supplement: Supplementary file 1 — The hearing thresholds of air conduction at 0.25, 0.5, 1, 2, 4, and 8 kHz were measured and adjusted (Supplementary Table 1). The air (0.25–8 kHz) and bone (0.5–4 kHz) conduction hearing deterioration with and without age-related correction at the 1- to 9-year follow-up after RT were listed in Supplementary Table 2 and 3, respectively. The mean air and bone conduction hearing deterioration at 0.5–4 kHz with and without age-related correction were measured (Supplementary Table 4). [file 769806.f1.pdf]

Supplementary TABLE 1: The air conduction hearing threshold at 0.25–8 kHz with and without age-related correction at the 1- to 9-year follow-up after RT. (Mean±SEM)

Years	0.25K Hz	0.5K Hz	1K Hz	2K Hz	4K Hz	8K Hz
**Initial year**						
**With age-adjusted**	23.03±1.15	21.81±1.13	24.71±1.38	25.34±1.53	27.97±1.60	26.63±1.53
**Without age-adjusted**	26.13±1.21	25.66±1.19	29.23±1.48	32.53±1.70	42.57±2.00	48.02±2.10
**First year**						
**With age-adjusted**	33.22±2.35	32.71±2.60	33.81±2.63	34.11±2.87	41.18±2.84	40.84±2.79
**Without age-adjusted**	36.46±2.39	36.71±2.66	38.10±2.75	41.58±3.11	58.61±3.25	62.67±3.44
**Second year**						
**With age-adjusted**	36.27±2.54	33.87±2.53	36.54±2.73	35.73±2.93	40.76±2.87	42.17±2.75
**Without age-adjusted**	39.46±2.57	37.89±2.58	41.27±2.82	43.31±3.03	57.35±3.21	65.66±3.38
**Third year**						
**With age-adjusted**	34.275±3.12	31.804±3.08	35.657±3.28	35.794±3.21	42.725±3.71	48.176±3.85
**Without age-adjusted**	37.75±3.18	35.98±3.16	41.08±3.39	43.92±3.42	59.22±4.24	71.18±4.49
**Fourth year**						
**With age-adjusted**	41.83±2.51	38.92±2.42	42.1±2.67	43.25±2.67	48.38±3.17	51.56±2.81
**Without age-adjusted**	45±2.55	43.05±2.48	47.29±2.78	51.72±2.81	65.52±3.57	76.38±3.13

**Fifth year**						
**With age-adjusted**	42.79±3.07	42.85±3.00	45.75±3.19	45±3.42	49.24±3.49	54.76±3.49
**Without age-adjusted**	46.12±3.26	47.16±3.18	50.75±3.34	52.99±3.66	66.04±3.81	79.25±3.43
**6th – 7th years**						
**With age-adjusted**	51.6±3.16	49.45±3.29	49.97±3.32	51.85±3.30	60.21±3.58	59.09±3.33
**Without age-adjusted**	55.47±3.2	54.20±3.46	56.70±3.48	61.33±3.68	77.37±3.87	83.39±3.44
**8th – 9th years**						
**With age-adjusted**	66.04±4.7	61.1±4.80	58.82±5.28	60.37±5.24	63.57±5.94	58.74±4.91
**Without age-adjusted**	69.40±4.8	66.00±5.05	64.63±5.43	70.10±5.58	84.94±6.27	89.81±5.62

* SEM = standard error of the mean

Supplementary TABLE 2: The air conduction hearing deterioration at 0.25–8 kHz with and without age-related correction at the 1- to 9-year follow-up after RT. (Mean±SEM)

Years	0.25K Hz	0.5K Hz	1K Hz	2K Hz	4K Hz	8K Hz
**First year**						
**With age-adjusted**	8.17±1.61	10.25±1.74	7.66±1.49	5.87±1.64	11.05±2.21	13.63±2.58
**Without age-adjusted**	8.16±1.61	10.25±1.74	7.66±1.49	5.63±1.66	12.22±2.12	13.56±3.2
**Second year**						
**With age-adjusted**	13.68±2.12	12.71±2.00	11.67±2.18	10.65±2.08	12.31±2.25	14.35±2.46
**Without age-adjusted**	13.68±2.12	12.71±2.00	11.69±2.18	10.72±2.09	14.8±2.16	16.45±2.34
**Third year**						
**With age-adjusted**	13.96±2.8	12.78±2.8	12.15±2.68	12.74±2.58	17.59±3.25	23.22±3.91
**Without age-adjusted**	14.12±2.79	13.04±2.80	12.84±2.66	13.24±2.55	20.69±3.09	27.94±3.68
**Fourth year**						
**With age-adjusted**	18.9±2.04	16.49±2.09	15.56±2.18	16.41±2.08	19.16±2.83	23.29±2.63
**Without age-adjusted**	19.08±2.02	16.90±2.07	16.08±2.17	17.99±2.05	22.74±2.83	27.53±2.45
**Fifth year**						
**With age-adjusted**	21.96±2.81	22.40±2.75	21.93±2.86	20.31±2.93	23.40±3.16	27.58±3.78
**Without age-adjusted**	22.61±3.03	23.28±2.95	23.06±3.05	22.24±3.11	26.57±3.29	32.16±3.56

**6th – 7th years**						
**With age-adjusted**	30.75±3.04	30.4±2.95	27.27±3.05	31.11±3.15	36.50±3.36	30.60±3.41
**Without age-adjusted**	31.65±3.17	31.46±3.06	29.98±3.12	33.7±3.25	41.22±3.53	39.23±3.43
**8th – 9th years**						
**With age-adjusted**	47.25±4.82	42.91±5.33	38.05±5.88	40.6±5.23	43.38±5.99	30.80±6.11
**Without age-adjusted**	47.94±4.80	44.34±5.3	40.69±5.67	44.71±5.28	52.84±5.93	43.21±6.18

* SEM = standard error of the mean

Supplementary TABLE 3: The bone conduction hearing deterioration at 0.5–4 kHz with and without age-related correction at the 1- to 9-year follow-up after RT. (Mean±SEM)

Years	0.5K Hz	1K Hz	2K Hz	4K Hz
**First year**				
**With age-adjusted**	4.96±1.33	2.77±0.92	4.09±1.21	6.68±1.73
**Without age-adjusted**	4.81±1.35	2.58±0.95	3.92±1.25	7.53±1.79
**Second year**				
**With age-adjusted**	7.06±1.62	6.69±1.27	5.00±1.38	4.74±1.36
**Without age-adjusted**	6.96±1.64	6.63±1.28	5.66±1.44	8.31±1.66
**Third year**				
**With age-adjusted**	6.00±1.69	6.35±1.77	6.45±1.68	7.32±2.2
**Without age-adjusted**	6.27±1.7	6.76±1.74	6.96±1.53	10.10±2.11
**Fourth year**				
**With age-adjusted**	10.47±1.41	10.43±1.42	9.32±1.34	9.97±1.82
**Without age-adjusted**	10.90±1.43	11.21±1.43	11.03±1.34	14.37±1.75
**Fifth year**				
**With age-adjusted**	12.78±1.8	11.67±1.9	12.84±2.19	11.34±2.46
**Without age-adjusted**	13.58±1.95	12.67±2.05	14.48±2.3	13.88±2.48

**6th – 7th years**				
**With age-adjusted**	19.60±2.22	21.71±2.33	21.09±2.12	20.69±2.48
**Without age-adjusted**	20.44±2.29	22.13±2.38	23.84±2.19	25.46±2.5
**8th – 9th years**				
**With age-adjusted**	28.73±3.54	28.72±3.98	27.95±3.82	23.97±4.41
**Without age-adjusted**	29.81±3.54	30.25±4.02	32.25±4.11	33.71±3.5

* SEM = standard error of the mean

Supplementary TABLE 4: The air and bone conduction hearing deterioration at 0.5–4 kHz with and without age-related correction at the 1- to 9-year follow-up after RT.

**Years**	**Air (dB) Mean±SEM**	**p value**	**Bone (dB) Mean±SEM**	**p value**
**First year**				
**With age-adjusted**	8.71±0.90	0.16	4.63±0.67	0.55
**Without age-adjusted**	8.94±0.89		4.71±0.69	
**Second year**				
**With age-adjusted**	11.84±1.06	0.02	5.87±0.71	0.001
**Without age-adjusted**	12.48±1.05		6.89±0.76	
**Third year**				
**With age-adjusted**	13.81±1.42	0.000	6.53±0.92	0.005
**Without age-adjusted**	14.95±1.40		7.52±0.89	
**Fourth year**				
**With age-adjusted**	16.91±1.16	0.000	10.05±0.75	0.000
**Without age-adjusted**	18.43±1.16		11.88±0.75	
**Fifth year**				
**With age-adjusted**	22.01±1.46	0.000	12.16±1.05	0.026

**Without age-adjusted**	23.79±1.55		13.65±1.10	
**6th – 7th years**				
**With age-adjusted**	31.57±1.57	0.000	20.55±1.14	0.000
**Without age-adjusted**	34.09±1.63		22.96±1.17	
**8th – 9th years**				
**With age-adjusted**	41.25±2.70	0.000	27.34±1.96	0.000
**Without age-adjusted**	45.64±2.74		31.5±1.88	

* SEM = standard error of the mean
